# Synergistic adverse prognosis of *CEBPA* and *NRAS* co-mutation in acute myeloid leukemia: a retrospective cohort study

**DOI:** 10.3389/fcell.2026.1775927

**Published:** 2026-03-18

**Authors:** Fujun Qu, Mingxu Hui, Fan Yang, Weiwei Wan, Nian Liu, Xianyi Wu, Yuchan He, Xiaotao Wang

**Affiliations:** 1 Department of Hematology, The First Affiliated Hospital of Guilin Medical University, Guilin, China; 2 Department of Radiation Oncology, The Second Affiliated Hospital of Guilin Medical University, Guilin, China; 3 Department of Pathology, The First Affiliated Hospital of Guilin Medical University, Guilin, China; 4 Health Management Center, The Second Affiliated Hospital of Guilin Medical University, Guilin, China; 5 Department of Hematology, The Second Affiliated Hospital of Guilin Medical University, Guilin, China

**Keywords:** acute myeloid leukemia, CEBPA, co-mutation, molecular subtype, NRAS, prognosis

## Abstract

**Background:**

Current prognostic stratification for acute myeloid leukemia (AML) patients primarily relies on international guidelines such as ELN 2022. However, these guidelines provide limited explicit guidance on how to comprehensively assess prognosis when patients harbor concurrent multiple gene mutations, potentially leading to inaccurate risk stratification.

**Methods:**

This retrospective cohort study enrolled 299 adult AML patients (median age: 53.5 years). Clinical features, mutation status of prognosis-related genes, complete remission (CR) rate after two induction courses, and overall survival (OS) were analyzed. Logistic regression and Cox proportional hazards models were employed.

**Results:**

In this study cohort, the incidence rates of *NRAS* and CEBPA single mutations were 15.38% (46/299) and 13.37% (40/299), respectively, while the *NRAS* and *CEBPA* co-mutation rate was 4.68% (14/299). Both univariate and multivariate logistic regression analyses demonstrated that *CEBPA* mutation (OR = 2.807, 95% CI: 1.244–6.333, *P* = 0.013) and *NRAS* mutation (OR = 4.028, 95% CI: 1.760–9.219, *P* = 0.001) were independent positive predictive factors for achieving complete remission after two treatment courses. Survival analysis indicated that neither *NRAS* single mutation (HR = 1.11, 95% CI: 0.60–2.04, *P* = 0.738) nor *CEBPA* single mutation (HR = 0.70, 95% CI: 0.36–1.34, *P* = 0.280) was significantly associated with overall survival. Notably, *NRAS* and *CEBPA* co-mutation was confirmed as an independent adverse prognostic factor, conferring a 3.15-fold increased risk of death (HR = 3.15, 95% CI: 1.08–9.21, *P* = 0.036) compared to patients without either mutation.

**Conclusion:**

The *NRAS* and *CEBPA* co-mutation defines a distinct molecular subtype with independently poor prognosis in AML. Routine *NRAS* mutation screening in patients with *CEBPA*-mutated AML is warranted to refine risk stratification, particularly for identifying this high-risk subgroup, which may benefit from more intensive or novel therapeutic approaches.

## 1 Introduction

Acute myeloid leukemia (AML) is a highly heterogeneous hematological malignancy characterized by significant prognostic variability, in which genetic alterations play a decisive role in disease classification, risk stratification, and treatment selection (Metzeler et al., 2016). Current clinical practice for prognostic assessment in AML primarily relies on international consensus guidelines such as ELN 2022 (Dohner et al., 2022). However, a significant challenge arises when patients carry multiple gene mutations simultaneously. Existing guidelines lack a clear framework for comprehensively evaluating the prognostic impact of such co-mutations, which may lead to biases in risk stratification and suboptimal treatment decisions (Boscaro et al., 2023; Loghavi et al., 2024). It is increasingly recognized that gene co-mutations often influence disease progression through complex synergistic mechanisms rather than simple additive effects (Shih et al., 2015; Mupo et al., 2013; Yao et al., 2024; Shahid et al., 2020). Interactions between different mutational pathways may alter key biological processes such as cell proliferation, differentiation, apoptosis, and signal transduction, thereby collectively shaping a more aggressive disease phenotype and contributing to poor clinical outcomes (Song et al., 2024; Ruhnke et al., 2025; Rausch et al., 2023). For instance, co-mutation patterns involving genes like *NPM1* and *FLT3-ITD* or *RUNX*1 and *ASXL1* have been shown to confer distinct prognostic implications beyond those of the individual mutations (Papaemmanuil et al., 2016).

Despite this, the specific prognostic interplay between mutations in key regulators like *NRAS* (a critical signal transducer) and *CEBPA* (a master regulator of myeloid differentiation) remains inadequately defined. In particular, the potential synergy between the concurrent disruption of myeloid differentiation (driven by *CEBPA* mutation) (Pabst et al., 2001; Reckzeh and Cammenga, 2010) and the constitutive activation of cellular proliferation (driven by *NRAS* mutation) (Johnson et al., 2014) may represent a high-risk biological state that is not adequately captured by current risk models. This represents a critical knowledge gap in AML prognostication.

To investigate this issue, this study conducted a retrospective cohort analysis involving 299 adult AML patients (median age: 51 years). By systematically collecting patients’ clinical data and genetic sequencing results, the study focused on analyzing the single and co-mutation status of prognosis-related genes, including *NRAS* and *CEBPA*, and evaluated their correlations with the complete remission rate after two courses of induction chemotherapy and overall survival.

## 2 Methods

### 2.1 Study design and patient cohort

This retrospective cohort study included 299 consecutive adult patients with newly diagnosed acute myeloid leukemia who received treatment at the First Affiliated Hospital of Guilin Medical University between January 2019 and January 2025. All patients underwent comprehensive MICM classification and risk stratification. Prior to enrollment, written informed consent was obtained from each participant, permitting the use of clinical and molecular data for research purposes. The study protocol was approved by the Ethics Review Committee of Guilin Medical University (202506020850) and conducted in accordance with established ethical standards.

### 2.2 Data collection and variables

Baseline clinical and laboratory data were systematically recorded. Demographic information included sex and age. Patients were dichotomized into age groups of <51 and ≥51 years. The cutoff of 51 years was selected as it represents the median age of the entire cohort, providing balanced group sizes for statistical comparison and aligning with common age stratification approaches in AML research. Disease characteristics comprised French-American-British (FAB) subtype and genetic risk stratification according to the ELN 2022 criteria. FAB subtype was recorded as it was routinely reported in the diagnostic records during the patient enrollment period. While acknowledging that contemporary AML diagnosis and classification rely on the integrated WHO/ICC systems incorporating genetic features, the FAB classification is retained here to provide a consistent morphological description of the cohort and to align with historical data reporting practices within our institution. Prognosis-related gene mutations—including *NRAS*, *CEBPA*, *FLT3*, *NPM1*, and *TP53*—were assessed by next-generation sequencing, with a variant allele frequency cutoff of ≥2% considered positive. Treatment details were documented, differentiating between intensive “3 + 7” regimens and venetoclax-based low-intensity regimens. Complete remission (CR) status was evaluated after two cycles of standard induction therapy. Overall survival (OS) was used to​ measure the survival outcomes.

### 2.3 Next-generation sequencing (NGS) analysis

Genomic DNA was extracted from bone marrow aspirates obtained at diagnosis. A targeted sequencing panel encompassing the exons and key intronic regions of genes recurrently mutated in AML (including but not limited to *NRAS*, *CEBPA*, *FLT3*, *NPM1*, *WT1*, *TP53*, *IDH1/2*, *DNMT3A*, *RUNX1*, *JAK2*, etc.) was used. Library preparation was performed using the KAPA HyperPlus Kit, followed by sequencing on an Illumina NovaSeq 6000 platform with an average coverage depth of >1000x. Raw sequencing data (FASTQ files) were processed using a standard bioinformatics pipeline which we described previously (Yang et al., 2025).

A variant was considered positive and subjected to further analysis if it met the following criteria: (a) variant allele frequency (VAF) ≥2%; (b) not present in population databases (gnomAD allele frequency <0.1%) or documented as a known pathogenic germline variant; (c) predicted to have a damaging effect on the protein (e.g., nonsense, frameshift, splice-site, or missense mutations predicted as damaging by multiple *in silico* tools like SIFT, Polyphen-2).

The VAF cutoff of ≥2% was selected based on established sensitivity thresholds for detecting clonal hematopoietic mutations in AML using deep targeted sequencing, which balances high detection sensitivity with technical noise filtration (Jennings et al., 2017; Duncavage et al., 2021). *CEBPA* mutations were analyzed for both single (mono-allelic) and double (bi-allelic) mutations, as defined by the presence of one or two distinct pathogenic variants within the gene, respectively. The pathogenic potential of all variants, including those in *WT1*, was assessed by integrating evidence from population frequency, *in silico* prediction tools, and existing literature linking specific alterations to AML pathogenesis (Papaemmanuil et al., 2016; Richards et al., 2015).

### 2.4 Treatment regimens

Treatment regimens were stratified based on the patient’s ability to tolerate intensive chemotherapy, as assessed by age, performance status, and comorbidities: Patients eligible for intensive chemotherapy received one of the following standard “3 + 7”regimens: Cytarabine (100–200 mg/m^2^/day for 7 days) combined with idarubicin (12 mg/m^2^/day for 3 days) or daunorubicin (60–90 mg/m^2^/day for 3 days); or A regimen containing homoharringtonine (2 mg/m^2^/day for 7 days) and daunorubicin (40 mg/m^2^/day for 3 days), with cytarabine administered at 100 mg/m^2^/day for the first 4 days and 1 g/m^2^ every 12 h on days 5, 6, and 7. Patients’ ineligible for or refusing intensive chemotherapy received a venetoclax-based regimen: Venetoclax (100 mg on day 1, 200 mg on day 2, and 400 mg daily from day 3 to day 28) combined with a hypomethylating agent: either azacitidine (75 mg/m^2^/day for 7 days) or decitabine (20 mg/m^2^/day for 5 days).

### 2.5 Study definitions

Complete remission was defined as bone marrow blasts <5%, recovery of peripheral blood counts, and absence of extramedullary disease following two cycles of induction therapy. A gene mutation was defined by a variant allele frequency ≥2% on next-generation sequencing; co-mutation referred to the presence of two or more concurrent mutations. Progression-free survival was measured from treatment initiation to disease progression or last follow-up, and overall survival from treatment initiation to death from any cause or last follow-up.

### 2.6 Statistical analysis

Statistical analyses were performed using R software. Univariate logistic regression was first applied to identify factors associated with complete remission; variables with *P* < 0.05 were entered into a multivariate logistic regression model. Survival curves were generated using the Kaplan–Meier method and compared with the log-rank test. To assess independent prognostic factors for overall survival, univariate Cox regression was performed initially. Significant variables (*P* < 0.05) from the univariate analysis, along with biologically relevant factors including CEBPA and *NRAS* mutation status and an interaction term, were incorporated into a multivariate Cox proportional hazards model. Adjustments were made for age, sex, ELN 2022 risk category, and treatment intensity. All tests were two-tailed, with *P* < 0.05 considered statistically significant. Forest plots and survival curves were produced using the forestplot and survminer packages, respectively.

## 3 Results

### 3.1 Baseline clinical and molecular characteristics of the study cohort

**FIGURE 1:**
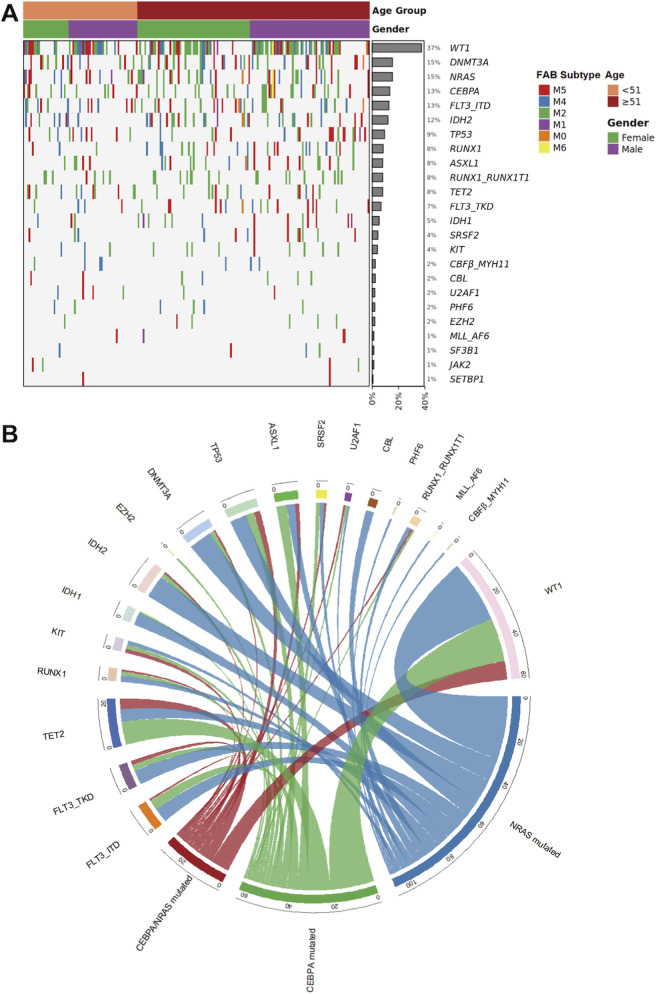
Baseline Characteristics and Mutational Landscape of the AML Cohort. **(A)** Heatmap depicting the co-occurrence patterns of gene mutations and their association with clinical features, including FAB subtype and co-mutation status. Each row represents a patient, and each column represents a gene or clinical variable. The mutational status (wild type vs. mutant) is color-coded. **(B)** The circos plot depicts pairwise co-occurrence of genetic mutations. In the outer ring, the width of each sector represents the total number of co-occurrences for that gene with the gene set on the opposite side. Sectors for the three primary genes (*NRAS* mutant, *CEBPA* mutant, and *CEBPA* and *NRAS* co-mutant) indicate co-occurrence with 22 other genes; sectors for the 22 associated genes indicate co-occurrence with the three primary genes. Inner ribbons connect co-mutated gene pairs, with ribbon width proportional to the co-occurrence strength.

The study cohort comprised 299 adult patients with newly diagnosed AML. Their median age was 53.5 years (IQR 42.8–58.3), with a near-balanced sex distribution (54.5% male). Morphologically, according to the FAB classification, the M2 subtype (36.3%) was predominant, followed by M5 (32.6%) and M4 (21.3%); the remaining categories (M1, M0, and M6) collectively represented less than 10% of cases. The distribution of FAB subtypes, along with the co-occurrence patterns of key gene mutations, is visually summarized in the heatmap (Figure 1A) (Table 1).

**TABLE 1:** Baseline characteristic of patients.

Characteristic	Age		P value
	<51 years (n = 110)	>51 years (n = 189)	
Male, n (%)	62 (56.4%)	101 (53.4%)	0.712
FAB type, n (%)			
M0	2 (1.8%)	1 (0.5%)	0.792
M1	6 (5.5%)	10 (5.3%)	
M2	41 (37.3%)	68 (36.0%)	
M4	25 (22.7%)	39 (20.6%)	
M5	36 (32.7%)	62 (32.8%)	
M6	0 (0%)	1 (0.5%)	
Mutation			
FLT3_ITD	13 (11.8%)	25 (13.2%)	0.863
FLT3_TKD	9 (8.2%)	21 (11.1%)	0.540
TET2	11 (10.0%)	13 (6.9%)	0.461
RUNX1	3 (2.7%)	22 (11.6%)	0.014
NPM1	14 (12.7%)	25 (13.2%)	1.000
KIT	3 (2.7%)	9 (4.8%)	0.576
CEBPA	12 (10.9%)	28 (14.8%)	0.435
IDH1	3 (2.7%)	13 (6.9%)	0.204
IDH2	8 (7.3%)	28 (14.8%)	0.080
EZH2	1 (0.9%)	5 (2.6%)	0.545
DNMT3A	13 (11.8%)	33 (17.5%)	0.255
TP53	10 (9.1%)	18 (9.5%)	1.000
ASXL1	5 (4.5%)	19 (10.1%)	0.142
JAK2	2 (1.8%)	2 (1.1%)	0.976
SRSF2	3 (2.7%)	10 (5.3%)	0.451
U2AF1	4 (3.6%)	2 (1.1%)	0.269
NRAS	14 (12.7%)	32 (16.9%)	0.421
CBL	2 (1.8%)	5 (2.6%)	0.952
SETBP1	1 (0.9%)	1 (0.5%)	1.000
ETV6	2 (1.8%)	1 (0.5%)	0.633
PHF6	2 (1.8%)	4 (2.1%)	1.000
SF3B1	1 (0.9%)	3 (1.6%)	1.000
RUNX1-RUNX1T1	7 (6.4%)	17 (9.0%)	0.557
MLL_AF6	1 (0.9%)	3 (1.6%)	1.000
CBFβ_MYH11	4 (3.6%)	3 (1.6%)	0.463
WT1	35 (31.8%)	77 (40.7%)	0.158
Clinical outcome			
CR, n (%)	58 (52.7%)	98 (51.9%)	0.884
DOD, n (%)	33 (30.0%)	93 (49.2%)	0.001
OS, Mean ± SD	529 ± 468	512 ± 471	0.767

Abbreviation: CR, complete remission; DOD, died of disease; OS, overall survival.

Genomic profiling identified distinct patterns of mutations. The *WT1*gene was the most frequently altered (37.3% of patients), while pathogenic variants in epigenetic regulators such as *DNMT3A* (15.3%) were also prevalent. Within signaling pathways, *NRAS* mutations were observed in 15.3% of patients. Notably, 4.7% of patients harbored a co-occurring *NRAS* and *CEBPA* mutation, delineating a molecular subgroup of particular interest. Other notable recurrent mutations included *NPM1*, F*LT3-ITD*, and *IDH2*, each with a frequency exceeding 12%. The pairwise co-occurrence of genetic mutations is additionally depicted in the circos plot (Figure 1B).

### 3.2 *CEBPA* and *NRAS* mutations independently predict treatment response

To identify molecular predictors of complete remission (CR) following two courses of induction therapy, univariate and multivariate logistic regression analyses were performed (Table 2). In univariate analysis, mutations in *CEBPA* (OR = 3.69, 95% CI: 1.69–8.06, *P* = 0.001), *NRAS* (OR = 5.43, 95% CI: 2.44–12.11, *P* < 0.001), and *WT1* (OR = 2.21, 95% CI: 1.36–3.57, *P* = 0.001) were each significantly associated with a higher likelihood of achieving CR. In contrast, other variables including age (≥51 years), sex, and mutations in *FLT3-ITD*, *FLT3-TKD*, *RUNX1*, *TP53*, *ASXL1*, *IDH1/2*, and *DNMT3A* showed no significant association in univariate testing (all *P* > 0.05).

**TABLE 2:** Univariate and multivariate analysis of CR after two induction courses in AML patients.

Variables	Univariate analysis		Multivariate analysis	
	OR (95% CI)	P value	OR (95% CI)	P value
Gender (female)	1.053 (0.668–1.668)	0.824	​	​
Age ≥51 years	0.966 (0.603–1.546)	0.884	​	​
FLT3_ITD	1.675 (0.830–3.382)	0.150	​	​
FLT3_TKD	1.130 (0.454–2.811)	0.793	​	​
TET2	0.910 (0.395–2.096)	0.824	​	​
RUNX1	0.992 (0.437–2.252)	0.985	​	​
NPM1	0.671 (0.341–1.331)	0.252	​	​
KIT	0.444 (0.131–1.508)	0.193	​	​
CEBPA	3.692 (1.691–8.064)	**0.001**	2.807 (1.244–6.333)	**0.013**
IDH1	0.699 (0.254–1.930)	0.490	​	​
IDH2	1.729 (0.840–3.559)	0.137	​	​
EZH2	0.451 (0.081–2.502)	0.363	​	​
DNMT3A	0.734 (0.391–1.379)	0.337	​	​
TP53	1.735 (0.773–3.895)	0.182	​	​
ASXL1	1.584 (0.671–3.741)	0.294	​	​
JAK2	2.784 (0.286–27.077)	0.378	​	​
SRSF2	1.073 (0.352–3.271)	0.902	​	​
U2AF1	0.451 (0.081–2.502)	0.363	​	​
NRAS	5.434 (2.438–12.112)	**0.000**	4.028 (1.760–9.219)	**0.001**
CBL	1.228 (0.270–5.584)	0.790	​	​
SETBP1	0.916 (0.057–14.784)	0.951	​	​
PHF6	0.915 (0.182–4.608)	0.914	​	​
SF3B1	0.916 (0.127–6.587)	0.930	​	​
RUNX1-RUNX1T1	0.759 (0.328–1.752)	0.518	​	​
MLL_AF6	0.916 (0.127–6.587)	0.930	​	​
CBFβ_MYH11	5.680 (0.675–47.768)	0.110	​	​
WT1	2.207 (1.363–3.574)	**0.001**	1.722 (1.034–2.867)	**0.037**
CEBPA and NRAS co-mut	12.91 (1.67–99.99)	**0.014**	1.39 (0.13–14.94)^a^	0.787^a^

^a^ When the CEBPA mutation, NRAS mutation, and the “CEBPA and NRAS, co-mutation” were all included in the multivariate analysis, the “CEBPA and NRAS, co-mutation” variable exhibited significant multicollinearity with the former two, resulting in a P-value <0.05. However, when only the “CEBPA and NRAS, co-mutation” and the WT1 mutation were included in the multivariate analysis model, the “CEBPA and NRAS, co-mutation” demonstrated robust independent predictive value, with an odds ratio (OR) = 11.08, 95% confidence interval (CI) = 1.41–86.73, and P = 0.022. The WT1 mutation had an OR, 2.07, 95% CI, 1.27–3.38, P = 0.004. Bold means significant difference.

Abbreviation: FLT3_ITD, Fms-like tyrosine kinase 3 - Internal Tandem Duplication, FLT3_TKD:Fms-like tyrosine kinase 3 - Tyrosine Kinase Domain mutation, TET2, Tet methylcytosine dioxygenase 2; RUNX1:Runt-related transcription factor 1, CEBPA:CCAAT/enhancer binding protein alpha, NPM1:Nucleophosmin 1, WT1, Wilms tumor 1; KIT, KIT, proto-oncogene, receptor tyrosine kinase; JAK2, Janus kinase 2; NRAS, Neuroblastoma RAS, viral oncogene homolog; CBL, Casitas B-lineage lymphoma proto-oncogene; IDH1, Isocitrate dehydrogenase 1; IDH2, Isocitrate dehydrogenase 2; EZH2, Enhancer of zeste 2 polycomb repressive complex 2 subunit; DNMT3A, DNA, methyltransferase 3 alpha; ASXL1, Additional sex combs like 1, transcriptional regulator; TP53, Tumor protein p53; SRSF2, Serine and arginine rich splicing factor 2; U2AF1, U2 small nuclear RNA, auxiliary factor 1; SF3B1, Splicing factor 3b subunit 1; SETBP1, SET, binding protein 1; ETV6, ETS, variant transcription factor 6; PHF6, PHD, finger protein 6; RUNX1-RUNX1T1, Runt-related transcription factor 1 - Runt-related transcription factor 1 translocated to 1; MLL_AF6, Mixed lineage leukemia - AF4/FMR2 family member 6; CBFβ-MYH11, Core-binding factor subunit beta - Myosin heavy chain 11.

After adjustment for age and cytogenetic risk in a multivariate model, mutations in *CEBPA* (OR = 2.81, 95% CI: 1.24–6.33, *P* = 0.013), *NRAS* (OR = 4.03, 95% CI: 1.76–9.22, *P* = 0.001), and *WT1* (OR = 1.72, 95% CI: 1.03–2.87, *P* = 0.037) remained independently associated with CR attainment. In contrast, other variables including age (≥51 years), sex, and mutations in *NPM1*, *FLT3-ITD*, *FLT3-TKD*, *RUNX1*, *TP53*, *ASXL1*, *IDH1/2*, and *DNMT3A* showed no significant association with CR in univariate testing (Table 2).

We further evaluated the specific impact of the *CEBPA* and *NRAS* co-mutation status. An initial attempt to include the co-mutation variable alongside the individual *CEBPA* and *NRAS* mutations in a multivariate model revealed significant multicollinearity, rendering the effect estimate for the co-mutation in that combined model statistically unstable. Therefore, to obtain a valid and interpretable estimate for the *CEBPA* and *NRAS* co-mutation itself, a separate multivariate model was constructed, which included only the *CEBPA* and *NRAS* co-mutation and the *WT1* mutation as genetic predictors, while adjusting for age and cytogenetic risk. In this model, the *CEBPA* and *NRAS* co-mutation emerged as a strong independent predictor for achieving CR (OR = 11.08, 95% CI: 1.41–86.73, *P* = 0.022). The *WT1* mutation also retained its independent significance (OR = 2.07, 95% CI: 1.27–3.38, *P* = 0.004). Collectively, these results indicate that while individual *CEBPA* and *NRAS* mutations are robust predictors of CR, the *CEBPA* and *NRAS* co-mutation status itself carries a pronounced and independent positive association with treatment response, warranting its distinct consideration in prognostic models.

### 3.3 The *CEBPA* and *NRAS* co-mutation defines a distinct high-risk subgroup

**FIGURE 2:**
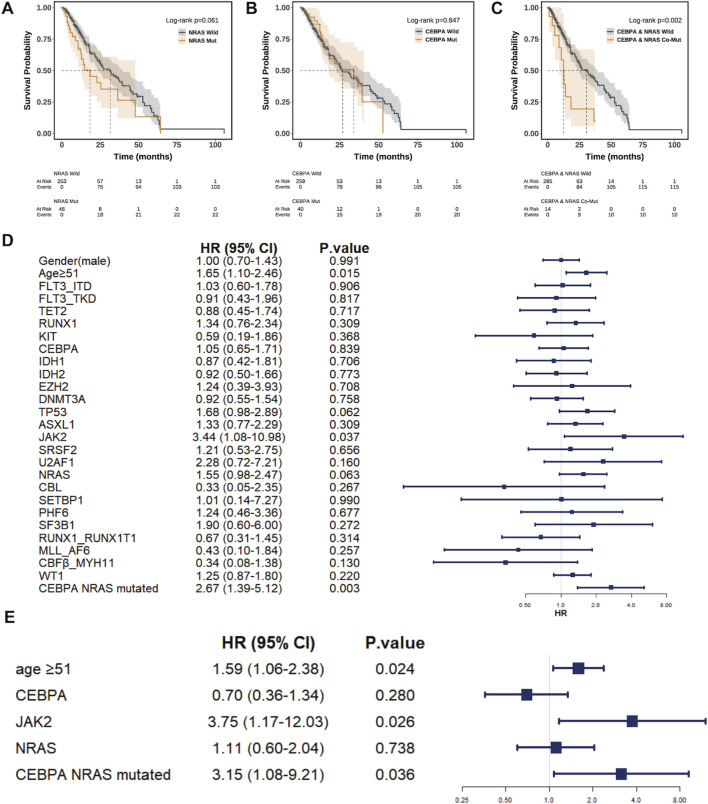
Impact of *NRAS* and *CEBPA* Mutations on Overall Survival. Kaplan-Meier curves comparing overall survival (OS) between AML patients with **(A)** *NRAS*-mut and NRAS wild, **(B)** *CEBPA*-mut and *CEBPA*-wild and **(C)** *NRAS* and *CEBPA* co-mutation and *NRAS* and *CEBPA* wild. Patients with co-mutation (*CEBPA*-mut and *NRAS*-mut) showed significantly inferior survival compared to the wild-type. **(D)** Forest plot of the univariate Cox analysis proportional hazards analysis for OS. Age ≥51 years, *JAK2* mutation, and the *CEBPA* and *NRAS* co-mutation are significant risk factors for mortality. Hazard ratios (HRs) and 95% confidence intervals are displayed. **(E)** Forest plot of the multivariate Cox proportional hazards analysis for OS. The model demonstrates the independent prognostic value of *CEBPA* mutation and its significant interaction with *NRAS* co-mutation. Hazard ratios (HRs) and 95% confidence intervals are displayed.

Kaplan-Meier survival analysis was performed to evaluate the impact of mutation status on overall survival (OS). Neither isolated *CEBPA* mutation (median OS: 34.6 vs. 27.3 months; *P* = 0.850) nor isolated *NRAS* mutation (median OS: 18.6 vs. 32.1 months; *P* = 0.061) showed a statistically significant effect on OS (Figures 2A,B). Contrary to this, patients harboring *CEBPA* and *NRAS* co-mutations exhibited a significantly inferior prognosis, with a median OS of only 12.6 months compared to 31.1 months in others (*P* = 0.002), suggesting a synergistic detrimental effect (Figure 2C).

We next conducted Cox regression analyses to identify independent prognostic factors. Univariate analysis confirmed age ≥51 years, *JAK2* mutation, and the *CEBPA* and *NRAS* co-mutation as significant risk factors for mortality (Figure 2D). Although isolated *CEBPA* and *NRAS* mutations were not individually significant for OS in univariate analysis, they were retained as predefined covariates in the multivariate model. This adjustment was based on their well-established biological relevance and their identified role as independent predictors of complete remission in our cohort. After adjusting for confounders, multivariate analysis confirmed that the *CEBPA* and *NRAS* co-mutation (HR = 3.15, 95% CI: 1.08–9.21, P = 0.036), along with age ≥51 years and *JAK2* mutation, were independent adverse prognostic factors. In this adjusted model, neither isolated *CEBPA* nor *NRAS* mutation retained independent prognostic value (Figure 2E).

## 4 Discussion

The refinement of molecular subtyping in AML has increasingly highlighted the prognostic influence of individual gene mutations, as well as the complex interplay between them (Dohner et al., 2022). Yet, the clinical implications of cooperative genetic events, particularly those involving differentiation and proliferation pathways, remain incompletely defined. Previous studies have underscored the importance of co-mutation patterns in AML prognosis. For example, research in specific populations and in *NPM1*-mutated AML has demonstrated that the co-mutational landscape significantly alters risk (Papaemmanuil et al., 2016). In this study, we focused on the prognostic interplay between *CEBPA* and *NRAS* mutations, with particular emphasis on the clinical and biological significance of their co-occurrence—a genotype not currently recognized in the ELN 2022 risk stratification framework.

We observed a seemingly paradoxical clinical pattern: while isolated *CEBPA* (OR = 2.807, *P* = 0.013) or *NRAS* (OR = 4.028, *P* = 0.001) mutation was independently associated with a higher likelihood of achieving CR, their co-mutation predicted significantly shorter overall survival (median OS 12.6 vs. 31.1 months, *P* = 0.002) and emerged as an independent adverse prognostic factor (HR = 3.15, *P* = 0.036). This dissociation between favorable initial treatment response and inferior long-term survival underscores that these two clinical endpoints can be decoupled in AML. While our cohort identified only 14 patients with the *NRAS* and *CEBPA* co-mutation—a subgroup size that warrants cautious interpretation—the findings suggest a distinct biological synergy. We hypothesize that the hyper-proliferative phenotype driven by mutant *NRAS* may confer sensitivity to intensive induction chemotherapy, leading to high CR rates (Johnson et al., 2014; Prior et al., 2012; Stone et al., 2017) 1. However, this initial response may be transient. The concomitant *CEBPA*-driven differentiation block and associated stem cell-like properties could establish a reservoir of therapy-persistent progenitor cells (Kirstetter et al., 2008; Fong et al., 2015), whose regeneration and expansion may be accelerated by the persistent proliferative signal from mutant *NRAS*, ultimately leading to early relapse and poor OS despite a high CR rate, a scenario observed in other aggressive AML subtypes (Fong et al., 2015; Vosberg and Greif, 2019).

This finding adds to a growing body of literature underscoring the complexity of risk assessment in AML (Wang et al., 2022; Li et al., 2021). In terms of initial treatment response, our data align with prior reports indicating that *CEBPA* mutation generally predicts favorable sensitivity to intensive chemotherapy, potentially through its role in promoting myeloid differentiation (Mao et al., 2024). The association between *NRAS* mutation and remission rates, however, has been inconsistent; while we observed improved response, other studies have reported lower remission or no significant effect (Dunna et al., 2014; Stein, 2018; Li et al., 2020). These discrepancies may reflect differences in patient populations, treatment intensity, or cooperating genetic contexts.

More critically, our long-term survival analysis did not support an independent adverse prognosis for either isolated *CEBPA* or *NRAS* mutation. In striking contrast, the presence of both mutations together exerted a marked synergistic effect, substantially increasing mortality risk relative to double-wild-type cases. This challenges the conventional risk-stratification paradigm that evaluates mutations in isolation and underscores the clinical relevance of co-mutational patterns, as also suggested by recent investigations (Boscaro et al., 2023; Bacher et al., 2008; Wang et al., 2016; Zhu et al., 2017; Zhou et al., 2022). The inferior OS associated with *CEBPA* and *NRAS* co-mutation might be attributable to a specific, aggressive mutational pattern or clonal architecture that promotes relapse and therapy resistance, a concept supported by studies investigating relapse-specific genetic patterns in AML (Vosberg and Greif, 2019). Consequently, therapeutic strategies specifically designed for patients with *CEBPA* and *NRAS* co-mutations are urgently needed.

The profound prognostic impact of the *CEBPA* and *NRAS* co-mutation revealed in our study is rooted in the distinct yet potentially convergent biological functions of these two genes in myeloid homeostasis and leukemogenesis. *CEBPA* encodes CCAAT/enhancer-binding protein alpha, a master transcription factor that is indispensable for granulocytic differentiation (Zhang et al., 1996). Biallelic *CEBPA* mutations, a defining feature in this cohort’s *CEBPA*-mutated cases, disrupt its normal function through complex molecular mechanisms including aberrant translation, protein misfolding, and dominant-negative effects, leading to a profound blockade in myeloid maturation and conferring a stem cell-like, self-renewal capacity to the leukemic clone, which forms the basis of the “differentiation arrest” phenotype in AML (Pabst et al., 2001; Reckzeh and Cammenga, 2010; Nerlov, 2004). Conversely, *NRAS* is a critical node in the RAS/MAPK signaling pathway, governing cellular proliferation, survival, and response to growth factors (Schubbert et al., 2007). Oncogenic *NRAS* mutations, predominantly at hotspots like G12, G13, and Q61, result in constitutive GTPase activity, leading to ligand-independent, hyperactive signaling that drives uncontrolled proliferation (Johnson et al., 2014; Prior et al., 2012). The co-occurrence of *CEBPA* and *NRAS* mutations, though individually not uncommon, represents a specific genotype whose combined biological and clinical impact remains poorly defined (Zhang et al., 2019).

The convergence of these two pathways—a differentiation block orchestrated by *CEBPA* dysfunction and a hyper-proliferative signal driven by mutant *NRAS*—may create a uniquely aggressive biological state. We hypothesize that the co-mutation does not merely additively combine two adverse features but may engender a synergistic loop. The *CEBPA*-driven differentiation arrest could expand a pool of immature, therapy-persistent progenitor cells (Kirstetter et al., 2008), while concurrent *NRAS* activation provides a potent proliferative signal to this pool, rapidly amplifying the resistant clone and fueling relapse (Amadori et al., 1981; Corces-Zimmerman et al., 2014). This model aligns with our clinical observation that the co-mutation strongly predicts early relapse and inferior OS, despite not being independently associated with lower initial CR rates. The latter may be explained by the retained sensitivity of the hyper-proliferative clone to intensive induction chemotherapy (Stone et al., 2017; Tomizawa et al., 2020), whereas the underlying differentiation block and stemness conferred by *CEBPA* mutation facilitate rapid regeneration of the leukemic population post-therapy (Fong et al., 2015), a process potentially accelerated by the constant proliferative drive of mutant *NRAS*. This mechanistic framework underscores why the *CEBPA* and *NRAS* co-mutation defines a molecular subset that warrants distinct risk stratification (Tomizawa et al., 2020). Importantly, while *NRAS* has been explored as a therapeutic target in other malignancies (Johnson et al., 2014), its co-mutation with *CEBPA* in AML presents a specific therapeutic challenge that warrants future functional studies to validate this pathogenic synergy and identify novel treatment strategies.

Beyond the core *CEBPA* and *NRAS* interplay, our multivariable model confirmed age ≥51 years and *JAK2* mutation as additional independent adverse prognostic factors. Older age integrates host- and disease-related vulnerabilities, while *JAK2* mutation aligns with previous reports of poor outcome linked to constitutive JAK-STAT signaling (Li et al., 2008; Michallet et al., 2011; Dai et al., 2022). Collectively, these results help delineate a higher-risk profile encompassing older age, activated signaling pathways such as *JAK2*, and the “differentiation-proliferation” dual hit of *CEBPA* and *NRAS* co-mutation. Such an integrated view may better inform risk-adapted therapeutic approaches.

Several limitations of our study warrant mention. Firstly, the retrospective, single-center design and the relatively small size of the co-mutation subgroup necessitate validation in prospective, multicenter cohorts. Secondly, mechanistic insights discussed here are largely inferred from the literature; future studies using gene-editing models are needed to functionally validate the proposed synergy between *CEBPA* and *NRAS*. From a translational standpoint, our findings argue for incorporating *CEBPA* and *NRAS* co-mutation into the next-generation of AML risk-stratification schemas. For this high-risk subset, rationale-based combination strategies—such as adding differentiation-inducing agents (e.g., retinoids) or downstream RAS and MAPK inhibitors (e.g., MEK inhibitors) to intensive chemotherapy—should be explored to concurrently target differentiation blockade and hyperactive proliferation, with the goal of improving long-term outcomes (Zhang et al., 2012). Thirdly, the study period (2019–2025) overlapped with the global COVID-19 pandemic, particularly its peak phases in 2020–2021. Although we have strived to maintain data integrity, the pandemic may have introduced unmeasured confounding factors, such as disruptions to standard treatment schedules, delays in diagnosis or follow-up, and increased mortality from non-leukemic causes (e.g., SARS-CoV-2 infection). These factors could potentially influence treatment response rates and overall survival outcomes, and their impact, though difficult to quantify, should be considered when interpreting the results. Furthermore, the use of the FAB classification system, though useful for morphological description, is recognized as an outdated framework that has been superseded by modern genetic-based classifications (WHO/ICC). This may limit the direct comparability of our cohort’s morphological distribution with studies using exclusively contemporary diagnostic criteria.

## 5 Conclusion

In summary, this study demonstrates that *CEBPA* and *NRAS* co-mutation exerts a significant synergistic adverse prognostic effect in AML, with a risk magnitude far exceeding that of individual mutations and can be integrated with traditional factors such as advanced age and *JAK2* mutation. These findings advocate for integrating co-mutation patterns, particularly the *CEBPA* and *NRAS* co-mutation, into future AML risk-stratification models to better identify high-risk patients and guide the development of tailored therapeutic strategies.

## Data Availability

The original contributions presented in the study are included in the article/supplementary material, further inquiries can be directed to the corresponding authors.
